# Retraction Note: LRRK2 kinase regulates α-synuclein propagation via RAB35 phosphorylation

**DOI:** 10.1038/s41467-026-77966-3

**Published:** 2026-10-05

**Authors:** Eun-Jin Bae, Dong-Kyu Kim, Changyoun Kim, Michael Mante, Anthony Adame, Edward Rockenstein, Ayse Ulusoy, Michael Klinkenberg, Ga Ram Jeong, Jae Ryul Bae, Cheolsoon Lee, He-Jin Lee, Byung-Dae Lee, Donato A. Di Monte, Eliezer Masliah, Seung-Jae Lee

**Affiliations:** 1 Departments of Biomedical Sciences and Medicine, Neuroscience Research Institute, Seoul National University College of Medicine, Seoul, 03080 Korea; 2 Molecular Neuropathology Section, Laboratory of Neurogenetics, National Institute on Aging, National Institutes of Health, Bethesda, MD 20892 USA; 3 Department Neurosciences, School of Medicine, University of California, San Diego, La Jolla, CA 92093 USA; 4 German Center for Neurodegenerative Diseases (DZNE), Sigmund-Freud-Strasse 27, 53127 Bonn, Germany; 5 Department of Neuroscience, Graduate School, Kyung Hee University, Seoul, 02447 Korea; 6 Department of Anatomy, School of Medicine, Konkuk University, Seoul, 05029 Korea; 7 Department of Physiology, School of Medicine, Kyung Hee University, Seoul, 02447 Korea; 8 Department of Pathology, School of Medicine, University of California, San Diego, La Jolla, CA 92093 USA

Retraction to: *Nature Communications* https://doi.org/10.1038/s41467-018-05958-z, published online 27 August 2018

The Authors have retracted this article. After publication, concerns were raised regarding some of the data presented in the Figures, specifically:

- Fig. 7a N2 image appears highly similar to Fig. 3a dat-16(mu86); V1S+SV2 (-) image in^1^;
- Fig. 8f p-ser129 a-syn C. Callosum Non-tg Veh and LKI images appear to overlap;
- Fig. 9d Non-tg LKI and α-synuclein tg Veh images appear to overlap.

Further checks by the Publisher found some highly similar patterns and other irregularities in the images presented in Fig. 8.

The Authors have stated that the quantitative analyses reported in the article were derived from the complete image datasets rather than from the representative panels displayed, and that the underlying source data are retained. The Authors nonetheless accept that the integrity of the figures as published cannot be assured, and they have therefore decided to retract the article.

Author Edward Rockenstein is deceased. All other authors agree to this retraction.
